# The Impact of Delayed Chemotherapy on Its Completion and Survival Outcomes in Stage II Colon Cancer Patients

**DOI:** 10.1371/journal.pone.0107993

**Published:** 2014-09-19

**Authors:** Fang Xu, Alfred A. Rimm, Pingfu Fu, Smitha S. Krishnamurthi, Gregory S. Cooper

**Affiliations:** 1 Department of Epidemiology and Biostatistics, Case Western Reserve University School of Medicine, Cleveland, Ohio, United States of America; 2 Department of Gastroenterology, University Hospitals Case Medical Center, Cleveland, Ohio, United States of America; 3 Division of Hematology and Oncology, Case Western Reserve University School of Medicine, Cleveland, Ohio, United States of America; 4 Case Comprehensive Cancer Center, Case Western Reserve University, Cleveland, Ohio, United States of America; University of Texas Southwestern Medical Center, United States of America

## Abstract

**Background:**

Delayed chemotherapy is associated with inferior survival in stage III colon and stage II/III rectal cancer patients, but similar studies have not been performed in stage II colon cancer patients. We investigate the association between delayed and incomplete chemotherapy, and the association of delayed chemotherapy with survival in stage II colon cancer patients.

**Patients and Methods:**

Patients (age ≥66) diagnosed as stage II colon cancer and received chemotherapy from 1992 to 2005 were identified from the linked SEER–Medicare database. The association between delayed and incomplete chemotherapy was assessed using unconditional and conditional logistic regressions. Survival outcomes were assessed using stratified Cox regression based on propensity score matched samples.

**Results:**

4,209 stage II colon cancer patients were included, of whom 73.0% had chemotherapy initiated timely (≤2 months after surgery), 14.7% had chemotherapy initiated with moderate delay (2–3 months), and 12.3% had delayed chemotherapy (≥3 months). Delayed chemotherapy was associated with not completing chemotherapy (adjusted odds ratio (OR): 1.33 (95% confidence interval: 1.11, 1.59) for moderately delayed group, adjusted OR: 2.60 (2.09, 3.24) for delayed group). Delayed chemotherapy was associated with worse survival outcomes (hazard ratio (HR): 1.75 (1.29, 2.37) for overall survival; HR: 4.23 (2.19, 8.20) for cancer-specific survival).

**Conclusion:**

Although the benefit of chemotherapy is unclear in stage II colon cancer patients, delay in initiation of chemotherapy is associated with an incomplete chemotherapy course and poorer survival, especially cancer-specific survival. Causal inference in the association between delayed initiation of chemotherapy and inferior survival requires further investigation.

## Introduction

Current guidelines from American Society of Clinical Oncology(ASCO) and National Comprehensive Cancer Network (NCCN) suggest chemotherapy use in stage III, high-risk stage II colon, and stages II and III colorectal cancer patients [1], [2]. However, the guidelines don’t specify the appropriate timing of chemotherapy initiation after surgery. In clinical trials, initiation ranged from 35 days [3] to 6 weeks [4], and intervals of longer than 8 weeks after surgery were associated with worse survival [5], [6]. In addition, a meta-analysis study suggested that delaying chemotherapy over 2 months after surgery could be deleterious to survival [7]. Another recent meta-analysis indicated a decrease in relative overall survival by 14% for every 4 weeks’ delay to initiation of chemotherapy [8].

There has been a dearth of research about delayed chemotherapy and the survival outcomes. To our knowledge, no large-database study has assessed the effect of delay in stage II colon cancer patients alone, given the fact that the effectiveness of chemotherapy is not as clear as stage III patients. Nonetheless, 20% of stage II patients still received post-surgical chemotherapy [9]. Due to ethical issues, it is infeasible to design a randomized clinical trial to study the impact of delayed therapy on survival and chemotherapy completion, and thus, well-designed retrospective studies are the best option to address this question.

## Materials and Methods

### Study population

The study utilized the linked Surveillance, Epidemiology, and End Results (SEER)– Medicare database. Detailed description of the database has been previously published [10], [11]. The study population included patients residing in the geographic areas served by SEER registries who were 66 years and older diagnosed with adenocarcinoma of the colon between 1992 and 2005, and were treated with surgical resection. Patients were excluded if they enrolled in Medicare who were disabled or had end-stage renal diseases, had a history of prior cancer, were diagnosed from autopsy or death certificate only, or had a second cancer diagnosis within 1 year after colon cancer diagnosis due to the possibility of affecting the use of chemotherapy. The study was limited to only patients with American Joint Committee on Cancer (AJCC) stage II. In order to ensure complete claims, patients were included if they were enrolled in Medicare Part A and B 12 months prior to and 18 months after the cancer or until death. During this enrollment period, patients were excluded if they enrolled in an Health Maintenance Organization (HMO). Furthermore, exclusion criteria included colon resection more than 3 months after cancer diagnosis, or death occurrence within 3 months of surgery. Surgical codes were identified using Medicare inpatient, outpatient and Carrier files [12], [13]. Lastly, patients who never received 5-FU based chemotherapy within 1 year of cancer diagnosis were excluded.

### Measures

Chemotherapy initiation after surgery was categorized into <2 months (timely), 2–3 months (moderately delayed) and ≥3 months (delayed), which is consistent with the time frame of previous SEER–Medicare studies [14], [15]. Completion of chemotherapy was defined as 5 subsequent consecutive months of chemotherapy in which there was at least 1 chemotherapy claim. In order to avoid misclassifying the treatment for cancer recurrence (CPT-4 code: 36246-7, 47120, 47122, 47125, 47130, 47370-1, 47380-2, 76362, 76394, 76490, 36260, 47100; ICD-9 procedure code: 50.20-2, 50.29, 50.3, 50.4; ICD-9 diagnosis code: 197.7, 197.0-3, 197.8, 198.3-5, 198.41, 198.45, 198.48, 198.51, 197.04, 197.08), the claims were only considered for the primary cancer treatment, which began with the first claim date for surgery or chemotherapy after diagnosis, and ended with the claim date after which there were 3 months with no colon cancer treatment, or a cancer recurrence, or 9 months after diagnosis whichever came first. By doing so, chemotherapy wouldn’t be misclassified for cancer recurrence. This algorithm was developed and validated using SEER–Medicare databases [16] and used by other Medicare studies [17]–[19]. The chemo agent was 5-FU based. ICD-9, CPT/HCPCS codes, and NDC (National Drug Code) are presented in the tables S1 and S2.

Based on the information obtained from the databases, time-to-event was from month of cancer diagnosis through 2002 for overall survival and through 2000 for cancer-specific survival, in order to achieve a complete 5-year survival of follow-up. Overall survival time was defined as the cancer diagnosis date till all-cause death or the end of the follow-up date, 12/31/2007. For cancer-specific survival, survival time was defined as the cancer diagnosis date till colon cancer-specific death or the end of the follow-up date, 12/31/2005. Patients were censored if they were alive at the end of the study period or died of causes other than colon cancer. For non-cancer-specific survival, patients were censored if they were alive at 12/31/2005 or died of colon cancer.

Patients’ demographics included age categories (66–69, 70–74, 75–79, 80–84, 85+), gender; race categorized as white, African American, and other; and marital status. Geographic regions were categorized as Midwest, Northeast, South, and West. Residential areas included metropolitan, suburban, and rural. Socioeconomic status (SES) included percentage of non-English speakers, percentage of persons with at least a high school diploma, and median income, all at census tract level. Cancer-related variables were obtained from SEER data, including T stage, tumor grade, presence of perforation or obstruction at disease presentation, and inadequately sampled lymph nodes (<12). Comorbid conditions were measured by Deyo-adapted Charlson index using the Klabunde rule-out algorithm [20]. Charlson conditions including neoplasm were excluded because of the correlation with the primary diagnosis of cancer. We searched the conditions from 1 year to 30 days prior to the cancer diagnosis to warrant preexisting conditions. The number of comorbidities was then categorized as 0, 1, 2, and ≥3. The other variables included hospitalization, oxygen use, and wheelchair use within 1 year prior to cancer diagnosis, length of hospital stay, and postsurgical hospital readmission.

### Analysis

For descriptive analysis, patients’ demographic and clinical information was compared among groups of timing of chemotherapy (timely, moderately delayed, delayed), using mean ± standard deviation if continuous variables were normally distributed, median with interquartile range if the continuous variables were not normally distributed, and frequencies with proportions if variables were categorical. The group comparison was based on Kruskal Wallis for continuous variables and Pearson χ^2^ tests for categorical variables. Logistic regressions were performed to predict delayed chemotherapy, adjusting for patients’ demographics, socioeconomics, and clinical variables. The association of timing of initiation of chemotherapy with incompletion of chemotherapy was assessed by unadjusted and adjusted logistic regressions. To balance the characteristics of the chemotherapy treatment groups, and to reduce the selection bias by the factors that affected the timing of receiving chemotherapy, which inherently affected the treatment outcomes, we used a propensity score matching algorithm. The propensity score was applied to generate one-to-one matched samples, one matched sample for ≥3 months and <3 months, and the other for ≥2 months and <2 months, by all the aforementioned variables using one-to-one greedy matching technique [21]. Conditional logistic regressions were then fit to assess the association between timing of initiation and incompletion of chemotherapy. For survival analysis, propensity score was generated in the study sample to predict the likelihood of delayed chemotherapy (≥3 months) versus the others. One-to-one matching algorithm was then applied in the survival samples. Kaplan-Meier survival curves were generated for overall, cancer-specific, and non-cancer-specific survivals by timing of the chemotherapy groups. Stratified Cox models with strata of matching sets were constructed to compare the matched groups. We then assessed the likelihood of the modification of incomplete chemotherapy on survival. All analyses were performed using statistical software SAS 9.2 (SAS Institute, Inc., Cary, NC) and R 2.9.2 (R project for statistical computing).

This study was approved by the Institutional Review Board of University Hospital Case Medical Center.

## Results

The initial colon cancer sample included 194,546 patients. Patients were excluded sequentially if they had end stage renal disease (ESRD) or were disabled (6.2%), had a prior cancer diagnosis (7.7%), had histology other than adenocarcinoma (6.3%), developed any secondary cancer within 1 year of cancer diagnosis (2.9%), were diagnosed from autopsy or death certificate (0.2%), were diagnosed before 1992 (6.3%), were younger than 66 years old (14.5%), had incomplete Medicare or HMO enrollment (51.7%), had stages other than AJCC stage II (65.1%), didn’t undergo surgery within 3 months after cancer diagnosis (3.3%), had missing covariates of interest (2.3%), and received preoperative chemotherapy or radiation therapy or died within 3 months of surgery (0.3%). Finally, patients not receiving 5-FU based chemotherapy within 1 year of cancer diagnosis were excluded from the sample (78.3%). The final sample included 4,209 patients.

Patient characteristics according to the timing of chemotherapy (timely: 73.0%, moderately delayed: 14.7%, and delayed: 12.3%) are shown in Table 1. The median age was 73.6 years with the interquartile range of 69.8 and 77.6 (results not shown), 84.9% were white, and 5.2% were black. Compared to others, patients who received timely chemotherapy were more likely to be younger, white, and residing in the Midwestern US. Patients’ diagnosis later than 2002 was more likely to be associated with the delayed group. SES at census tract had no association with the timing of chemotherapy initiation. Other clinical characteristics associated with timely initiation were lower Charlson score, and no postsurgical hospital readmission. High-risk prognostic factors were not associated with timing of initiation. The results of logistic regressions predicting delayed chemotherapy are presented in Table 2. Age, SEER region, and readmission to the hospital were strong predictors. The other variables associated with delayed chemotherapy were African American race, a colon cancer diagnosis after 2002, length of hospital stay ≥14 days, and a Charlson comorbidity score of 2.

**Table 1:** Patient characteristics by the time of chemotherapy initiation.

Characteristics	All sample4,209 (100%)N(%)	Chemotherapyinitiated ≤2 monthsfrom surgery (timely)3,071 (73.0%) N(%)	Chemotherapy initiated2–3 months from surgery(moderately delayed) 619(14.7%) N(%)	Chemotherapyinitiated≥3 months fromsurgery (delayed)519 (12.3%) N(%)	p-value
**Demographics**					
Age at diagnosis					<0.001
66–69	1,113 (26.5)	845 (27.5)	162 (26.2)	106 (20.4)	
70–74	1,394 (33.1)	1,032 (33.6)	226 (36.5)	136 (26.2)	
75–79	1,141 (27.1)	828 (27.0)	157 (25.4)	156 (30.1)	
80–84	442 (10.5)	306 (10.0)	Note	79 (5.2)	
85+	119 (2.8)	60 (1.9)	Note	42 (8.1)	
Gender					0.95
Female	2,255 (53.6)	1,646 (53.6)	334 (54.0)	275 (53.0)	
Male	1,954 (46.4)	1,425 (46.4)	285 (46.0)	244 (47.0)	
Race					0.01
White	3,575 (84.9)	2,638 (85.9)	509 (82.2)	428 (82.5)	
Black	219 (5.2)	139 (4.5)	42 (6.8)	38 (7.3)	
Others	415 (9.9)	294 (9.6)	68 (11.0)	53 (10.2)	
Marital status					0.06
Married	2,585 (61.4)	1,921 (62.6)	372 (60.1)	292 (56.3)	
Not married	1,503 (35.7)	1,061 (34.5)	Note	Note	
Unknown	121 (2.9)	89 (2.9)	Note	Note	
SEER regions					<0.001
Midwest	1,072 (25.5)	834 (27.2)	141 (22.8)	97 (18.7)	
Northeast	846 (20.1)	579 (18.8)	141 (22.8)	126 (24.3)	
South	615 (14.6)	441 (14.4)	87 (14.0)	87 (16.7)	
West	1,676 (39.8)	1,217 (39.6)	250 (40.4)	209 (40.3)	
Residential places					0.30
Metropolitan	3,529 (83.8)	2,559 (83.3)	522 (84.3)	448 (86.3)	
Urban	599 (14.2)	456 (14.9)	Note	Note	
Rural	81 (1.9)	56 (1.8)	Note	Note	
**SES at census tract**					
Median income ($)	42,000 (31,570, 57,170)	41,708 (31,518, 56,454)	42,363 (31,900, 56,798)	42,476 (31,014, 60,367)	0.46
At least high schooldiploma (%)	83.6 (74.6, 90.3)	83.7 (74.9, 90.3)	83.0 (73.4, 89.9)	83.8 (73.6, 90.8)	0.44
Not well spokenEnglish at age 65+ (%)	1.5 (0, 5.1)	1.4 (0, 4.8)	1.8 (0, 5.7)	1.8 (0, 5.5)	0.03
Year of diagnosis					<0.001
1992–1995	923 (21.9)	722 (23.5)	97 (15.7)	104 (20.0)	
1996–1999	909 (21.6)	685 (22.3)	131 (21.1)	93 (17.9)	
2000–2002	1,366 (32.5)	998 (32.5)	229 (37.0)	139 (26.8)	
2003–2005	1,011 (24.0)	666 (21.7)	162 (26.2)	183 (35.3)	
**High-risk prognostic factors**					
T4	817 (19.4)	591 (19.2)	125 (20.2)	101 (19.5)	0.86
Poorly differentiated orundifferentiated tumor grade	813 (19.3)	585 (19.1)	119 (19.2)	109 (21.0)	0.58
Obstruction	271 (6.4)	194 (6.3)	40 (6.5)	37 (7.1)	0.78
Perforation	203 (4.8)	138 (4.5)	32 (5.2)	33 (6.4)	0.17
<12 lymph node examined	2,215 (52.6)	1,599 (52.1)	324 (52.3)	292 (56.3)	0.21
**Pre-diagnostic conditions**					
Charlson Index					0.002
0	3,033 (72.1)	2,256 (73.5)	429 (69.3)	348 (67.0)	
1	813 (19.3)	584 (19.0)	123 (19.9)	106 (20.4)	
2	252 (6.0)	159 (5.2)	Note	Note	
3+	111 (2.6)	72 (2.3)	Note	Note	
Hospitalization	553 (13.1)	393 (12.8)	85 (13.7)	75 (14.5)	0.53
Oxygen or wheelchair use	60 (1.4)	38 (1.2)	Note	Note	0.21
**Post-surgical conditions**					
Length of hospital stay					<0.001
≤14 days	3,768 (89.5)	2,832 (92.2)	512 (82.7)	424 (81.7)	
>14 days	441 (10.5)	239 (7.8)	107 (17.3)	95 (18.3)	
Hospital readmission					<0.001
0	3,450 (82.0)	2,721 (88.6)	445 (71.9)	284 (54.7)	
1	607 (14.4)	322 (10.5)	138 (22.3)	147 (28.3)	
2+	152 (3.6)	28 (0.9)	36 (5.8)	88 (17.0)	

Note: Small numbers are suppressed.

**Table 2:** Association of patient characteristics with the odds of delayed chemotherapy (≥3 months).

Characteristics	Unadjusted OR (95% CI)	p-value	Adjusted OR (95% CI)	p-value
**Demographics**				
Age at diagnosis				
66–69 (ref)	1	–	1	–
70–74	1.03 (0.79, 1.34)	0.84	1.02 (0.77, 1.35)	0.90
75–79	1.51 (1.16, 1.20)	0.002	1.44 (1.09, 1.90)	0.01
80–84	2.07 (1.51, 2.83)	<0.001	1.76 (1.25, 2.48)	0.001
85+	5.18 (3.39, 7.93)	<0.001	3.55 (2.20, 5.71)	<0.001
Gender				
Female (ref)	1	–	1	–
Male	1.03 (0.86, 1.24)	0.77	1.03 (0.84, 1.27)	0.77
Race				
White (ref)	1	–	1	–
Black	1.54 (1.07, 2.22)	0.02	1.68 (1.10, 2.55)	0.02
Others	1.08 (0.79, 1.46)	0.64	1.16 (0.79, 1.69)	0.46
Marital status				
Married (ref)	1	–	1	–
Not married	1.30 (1.08, 1.58)	0.01	1.05 (0.84, 1.31)	0.68
Unknown	0.95 (0.53, 1.70)	0.85	0.74 (0.39, 1.41)	0.37
SEER regions				
Midwest (ref)	1	–	1	–
Northeast	1.76 (1.33, 2.33)	<0.001	1.41 (1.01, 1.95)	0.04
South	1.66 (1.22, 2.25)	0.001	1.71 (1.20, 2.42)	0.003
West	1.43 (1.11, 1.85)	0.01	1.32 (0.98, 1.78)	0.07
Residential places				
Metropolitan (ref)	1	–	1	–
Urban	0.77 (0.58, 1.02)	0.07	0.92 (0.66, 1.28)	0.62
Rural	1.08 (0.57, 2.06)	0.81	1.34 (0.65, 2.74)	0.43
**SES at census tract**				
Median income ($)^a^	1.03 (0.99, 1.07)	0.18	1.03 (0.97, 1.09)	0.39
At least high school diploma (%)	1.00 (0.99, 1.01)	0.88	1.01 (1.00, 1.02)	0.24
Not well spoken English at age 65+ (%)	1.005 (0.996, 1.014)	0.26	1.00 (0.99, 1.02)	0.76
Year of diagnosis				
1992–1995 (ref)	1	–	1	–
1996–1999	0.90 (0.67, 1.21)	0.47	0.79 (0.57, 1.09)	0.15
2000–2002	0.89 (0.68, 1.17)	0.41	0.66 (0.49, 0.89)	0.01
2003–2005	1.74 (1.34, 2.26)	<0.001	1.35 (1.00, 1.81)	0.05
**High-risk prognostic factors**				
T4	1.00 (0.80, 1.27)	0.98	0.89 (0.69, 1.15)	0.36
Poorly differentiated orundifferentiated tumor grade	1.13 (0.90, 1.41)	0.30	1.07 (0.83, 1.37)	0.60
Obstruction	1.13 (0.79, 1.62)	0.49	0.91 (0.61, 1.35)	0.62
Perforation	1.41 (0.96, 2.07)	0.08	0.97 (0.63, 1.50)	0.89
<12 lymph node examined	1.18 (0.98, 1.42)	0.08	1.19 (0.97, 1.45)	0.10
**Pre-diagnostic conditions**				
Charlson Index				
0 (ref)	1	–	1	–
1	1.16 (0.92, 1.46)	0.22	1.05 (0.81, 1.36)	0.72
2	1.72 (1.23, 2.42)	0.002	1.48 (1.01, 2.19)	0.05
3+	1.59 (0.96, 2.64)	0.07	1.07 (0.58, 1.95)	0.83
Hospitalization^b^	1.14 (0.87, 1.48)	0.34	0.88 (0.65, 1.21)	0.44
Oxygen or wheelchair use^b^	1.61 (0.83, 3.12)	0.16	0.98 (0.46, 2.10)	0.96
**Post-surgical conditions**				
Length of hospital stay				
≤14 days (ref)	1	–	1	–
>14 days	2.17 (1.69, 2.78)	<0.001	1.38 (1.03, 1.84)	0.03
Hospital readmission^c^				
0 (ref)	1	–	1	–
1	3.56 (2.85, 4.45)	<0.001	3.23 (2.56, 4.08)	<0.001
2+	15.33 (10.87, 21.62)	<0.001	13.26 (9.20, 19.12)	<0.001

a median income at census tract divided by $10,000 for ease of interpretation.

b measured within 1 year prior to cancer diagnosis.

c measured after surgery till chemotherapy initiation.

The unadjusted model showed that patients in the moderately delayed group had 36% higher odds of not completing chemotherapy than those in the timely group (Table 3). Patients in the delayed group had 2.72 times higher likelihood of not completing chemotherapy. After adjusting for the confounders, the results did not change appreciably. Using 3 months as a cutoff point of delayed chemotherapy, we conducted the propensity score matching analysis. The sample size was 964 in total, in other words, 482 pairs in patients undergoing chemotherapy within and after 3 months. For each matched sample, the distributions of the propensity scores had decent overlaps comparing the groups. Additionally, there was no statistical difference between the matched groups in terms of the patient characteristics, which indicates the appropriate use of propensity matching algorithm. The conditional logistic regression showed that patients in the delayed group had 2.45 times higher likelihood of not completing chemotherapy than their counterparts. When the cutoff was 2 months, patients initiating chemotherapy ≥2 months had 68% higher odds of not completing chemotherapy (n = 1,996).

**Table 3:** Association of timing of initiation and incompletion of chemotherapy.

Timing of initiation of chemotherapyafter surgery	Incompletion of chemotherapyOR (95% CI)	p-value
Unadjusted model (n = 4,209)		
≤2 months (reference)	1	–
2–3 months	1.36 (1.14, 1.61)	<0.001
≥3 months	2.72 (2.22, 3.33)	<0.001
Adjusted model (n = 4,209)^a^		
≤2 months (reference)	1	–
2–3 months	1.33 (1.11, 1.59)	0.002
≥3 months	2.60 (2.09, 3.24)	<0.001
Propensity score matched sample 1^b^		
<2 months (reference)	1	–
≥2 months	1.68 (1.40, 2.00)	<0.001
Propensity score matched sample 2^c^		
<3 months (reference)	1	–
≥3 months	2.45 (1.85, 3.24)	<0.001

a adjusted by age, gender, race, marital status, SEER regions, residential places, median income, % at least.

high school diploma, % not speaking English well or at all at 65 years and older at census tract level,

year of diagnosis, T4 lesions, poorly differentiated or undifferentiated tumor grade, obstruction at disease.

presentation, perforation at disease presentation, <12 lymph nodes examined, Charlson index,

hospitalization or oxygen, wheelchair use within 1 year prior to cancer diagnosis, length of hospital stay after surgery and number of readmission after surgery.

b propensity score matching 1∶1 on <2 months and ≥2 months with covariates described above.

Conditional logistic regression was fit on the matched sample (n = 1,996).

c propensity score matching 1∶1 on <3 months and ≥3 months with covariates described above.

Conditional logistic regression was fit on the matched sample (n = 964).

For survival analysis, two samples were generated, one for overall survival (n = 3,198) and the other for cancer-specific survival (n = 2,320). For exploratory analysis purpose, the unadjusted Kaplan-Meier curves were plotted. There was an overall significant difference among the 4 strata (chemotherapy initiated <3 months and completed, chemotherapy initiated <3 months but not completed, chemotherapy initiated ≥3 months and completed, and chemotherapy initiated ≥3 months but not completed) for both overall and cancer-specific survivals (both log-rank tests: p-value<0.001, results not shown). Patients who initiated chemotherapy <3 months and completed the treatment had the best survival, those who had delayed chemotherapy (≥3 months) and didn’t complete chemotherapy regimen had the poorest survival. In addition, the timing of chemotherapy had a stronger impact on survival than incompletion of chemotherapy regimen.

**Figure 1:**
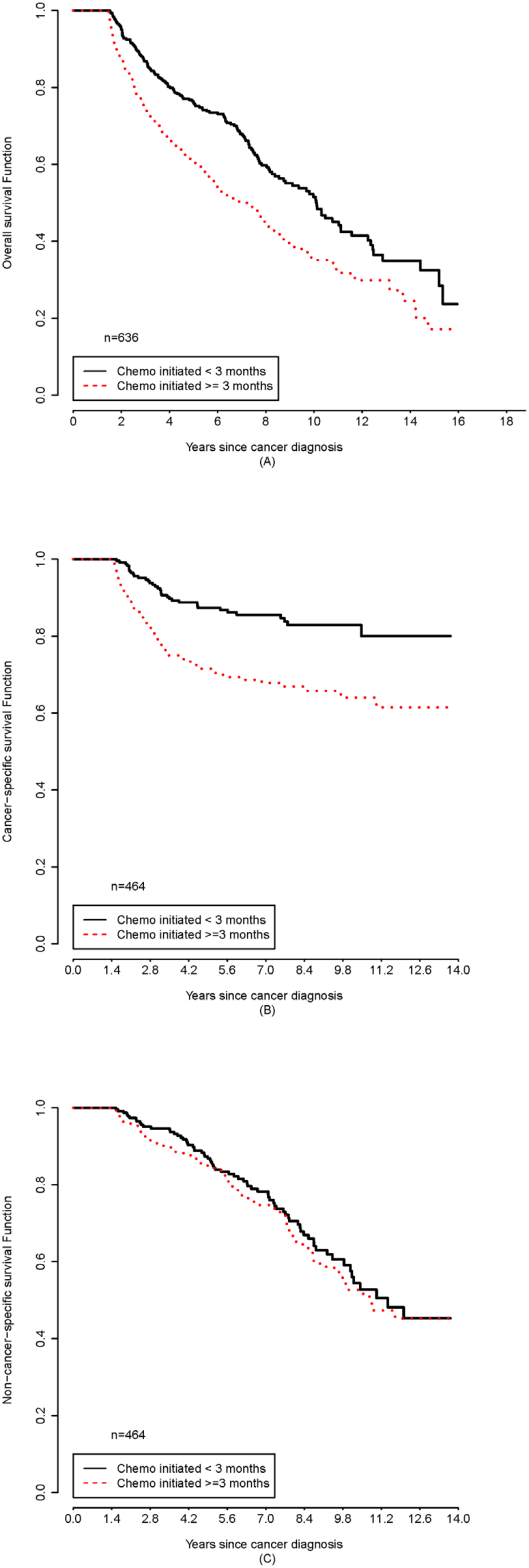
Kaplan-Meier curves for 3 matched samples from propensity score analysis. (A): matched sample (n = 636) for overall survival analysis. (B): matched sample (n = 464) for cancer-specific survival analysis. (C): matched sample (n = 464) for non-cancer-specific survival analysis. Due to the matched samples, Log-rank tests were not appropriate to generate p-values for group comparison. Therefore, p-values were not shown on the graphs. Group comparison was based on the models.

New samples were generated from the propensity score matching for overall survival (n = 636), non-cancer-specific survival (n = 464), and cancer-specific survival (n = 464) according to the delayed chemotherapy at the cutoff of 3 months. In the matched samples, the interaction term of delayed and incomplete chemotherapy was not significant, hence was not added in the stratified Cox regressions. Figure 1 shows the 3 Kaplan-Meier survival curves by timing of initiation (≥3 months and <3 months). Due to the design of the matched samples, Log-rank tests were not appropriate for the group comparison. Therefore, p-values were not shown on the graphs. Timely or moderately delayed group was associated with significantly better survival in overall and cancer-specific analyses, but the association was only borderline significant in non-cancer-specific survival. In the stratified Cox regressions taking pairs of matches into account, delayed group had significantly higher hazards of overall death (HR = 1.75; 95% CI (1.29, 2.37); p-value<0.001) and cancer-specific death (HR = 4.23; 95% CI (2.19, 8.20); p-value<0.001) than timely or moderately delayed group. The impact of delayed chemotherapy was much higher in cancer-specific survival. Incompletion of chemotherapy had no influence on survival (Table 4).

**Table 4:** Association of delayed chemotherapy (≥3 months) and survival in matched samples.

Variables	HR (95% CI)	p-value^b^
Overall survival (n = 636)		
Delayed chemotherapy^a^	1.75 (1.29, 2.37)	<0.001
Incomplete chemotherapy	1.32 (0.83, 2.11)	0.24
Colon-cancer-specific survival (n = 464)		
Delayed chemotherapy^a^	4.23 (2.19, 8.20)	<0.001
Incomplete chemotherapy	1.53 (0.60, 3.91)	0.38
Non-colon-cancer-specific survival (n = 464)		
Delayed chemotherapy^a^	1.69 (0.95, 3.01)	0.07
Incomplete chemotherapy	1.34 (0.59, 3.07)	0.49

a chemotherapy initiated ≥3 months after surgery, reference group is chemotherapy initiated <3 months after surgery.

b comparison based on stratified Cox regression.

Note: all models adjusted for age, gender, Charlson index, length of hospital stays and hospital readmission after surgery. The interaction terms of delayed chemotherapy modified by incomplete chemotherapy were not significant in all the models.

## Discussion

Very few studies have examined the predictors of delayed chemotherapy in stage II colon cancer patients because the survival benefit on chemotherapy is unclear in this population. Consistent with previous studies, we confirmed that older age, longer hospital stay after surgery, and hospital readmissions were strong predictors of delayed chemotherapy. Our results also showed that African Americans were more likely than the white to delay chemotherapy. Some literature suggested that African Americans were more likely to have an aversion to aggressive treatment or mistrust of the healthcare system or different interpretation of chemotherapy than white [22]–[24]. We found that regional variation and recent diagnosis were also associated with timing of initiation. It is unknown whether that reflected a true association or was driven by some hidden confounders that were not able to be controlled in the study. Very few studies have reported the associations of high-risk prognostic factors and initiation of chemotherapy. Our results revealed that some of these high-risk factors were not the predictors of initiation of chemotherapy. Rather, age, being black, and complications from surgery were.

In a study that investigated the length of interval and the survival outcomes in stage III colon cancer patients [14], patients who waited over 3 months to initiate chemotherapy had worse overall and cancer-specific survival than those who initiated chemotherapy within 3 months. Another study using stage II and III rectal cancer patients confirmed that those who delayed chemotherapy (≥3 months) had worse overall and cancer-specific survival [15], indicating that the delay of treatment was mainly influenced by the postoperative complications, hospital readmission, age and marital status. On the other hand, Dobie and the colleagues found that among the patients with stage III colon cancer who initiated chemotherapy in time, those who didn’t complete chemo regimen were older, being unmarried, had postsurgical complications and readmitted to hospital [16]. Another study indicates that patients with stage II and III rectal cancer who completed adjuvant therapy had better cancer-specific survival than those who didn’t complete therapy [17]. Some of the risk factors that are associated with the chemotherapy completion are also the factors that are associated with the initiation of chemotherapy. The relationship of treatment initiation and the completion of chemotherapy and the impact of the relationship on survival have not been well studied.

No study has examined the relationship between timing of initiation and completion of chemotherapy. Although delay and incompletion of chemotherapy shared similar characteristics, the association between delayed and incompletion of chemotherapy remained significant, even after controlling and balancing demographics and clinical variables. 5-FU based chemo agents usually have adverse outcomes such as diarrhea, leucopenia, and gastroenteritis [25]. Patients who delayed chemotherapy were generally sicker and thus were more likely to have poor tolerance of chemotoxicity. After the shared characteristics were adjusted, timing became an independent factor of completion of chemotherapy.

It was hypothesized that patients who had delayed and incomplete chemotherapy had worse survival. This was seen in the unadjusted analysis. In the matched samples, the incomplete chemotherapy was not a significant effect modifier, neither was it associated with survival outcomes. Instead, the delayed group was significantly associated with overall survival, especially with cancer-specific survival. Furthermore, the magnitude of the effect of delayed chemotherapy on non-cancer-specific survival was much smaller than that on cancer-specific survival, and the association was not significant, which could be implied that the significant association between delayed chemotherapy and overall survival was mostly driven by cancer-specific survival. Our results were inconsistent with a Canadian study where survival was affected by early discontinuation but not timing of adjuvant therapy [26]. First, our study population was different from theirs (stage III colon and stage II/III rectal cancer patients). Second, their end point was disease-free survival and overall survival. We were unable to assess disease-free survival because the database lacks date for relapse. Most importantly, our results were consistent with many previous studies analyzed in the meta-analyses [7], [8].

Although the benefit of chemotherapy is unclear in stage II colon cancer patients, we found that the impact of delayed chemotherapy on inferior survival was similar to stage III colon cancer patients where the role of chemotherapy was well established. Delayed adjuvant therapy causing deleterious survival was not only found in colorectal cancer [14], [15] but also in breast cancer [27]–[29]. Our results implied that the delay may be more likely to be related to cancer-specific survival. One early study showed that the effect of chemotherapy was diminished if its initiation was delayed [30]. A possible explanation is that tumor probably proliferates during the longer interval time. One study showed that the effect of chemotherapy decreased when the interval between surgery and initiation of chemotherapy increased based on experimental animals [31]. Although the benefit of chemotherapy is not obvious in stage II colon cancer patients, our results showed that timing rather than chemotherapy completion was more related to survival outcomes. We used propensity score matching algorithm to reduce bias. Delay itself seems to be an independent factor for adverse cancer-specific survival outcome. Therefore, the other possible explanation is that there are some biological factors of those patients who had delayed chemotherapy that were not able to be measured in the study that were inherently the causal contributors to the inferior survival rather than the single effect of chemotherapy. However, the biological pathway was unable to be determined in our study.

There are some limitations in the study. Patients were only limited to age older than 65 with fee-for-service insurance. Some of the clinical variables were unavailable due to the nature of the data, such as the actual dose and toxicity of the chemotherapy agents [32]. Nonetheless, this is the first population-based study to show the association between delayed and incomplete chemotherapy and the impact of delayed chemotherapy on inferior survivals among stage II colon cancer patients. During the study period, the use of new chemotherapy agents was uncommon among stage II colon cancer patients. We lacked sufficient power to study new agents in this study.

In summary, our study indicated delayed chemotherapy was associated with inferior overall and cancer-specific survivals. Future studies should focus on patients’ treatment preferences, social or physical support, ability of compliance, patient influence by physicians, and medical knowledge about adjuvant therapy which could add the contribution to the association between delayed and incomplete chemotherapy. In addition, future clinical investigation should focus on the tumor biology mechanism for patients who have delayed chemotherapy that could cause inferior survivals, especially cancer-specific survival.

## Supporting Information

File S1 Contains Table S1 and S2.
(DOCX)
