# Supplementary material for: The Impact of Delayed Chemotherapy on Its Completion and Survival Outcomes in Stage II Colon Cancer Patients
Source: PLoS One. 2014 Sep 19;9(9):e107993. doi: 10.1371/journal.pone.0107993 (PMC4169603; doi:10.1371/journal.pone.0107993)
Supplement: File S1 — Contains Table S1 and S2. (DOCX) [file pone.0107993.s001.docx]

Table S1: ICD-9, HCPCS, and NDC codes for chemotherapy

| Chemotherapy | | | | | |
| --- | --- | --- | --- | --- | --- |
| Administration | V58.1 | 99.25 | 0331, 0332, 0335 | Q0083-Q0085, 964xx, 965xx, G0355-G0363, 51720, |  |
| Specific agents |  |  |  | J9190, J0640, J9200, J9206, J9263, J9055, J8521, J8520 | 63759000, 000041100, 000041101, 548684143, 548685260 |
| Other | V66.2, V67.2 |  |  |  |  |

Table S2: Procedure codes for colon resection

| ICD-9 procedure codes | |
| --- | --- |
| Open and other partial excision of large intestine | 45.7 |
| Total intra-abdominal colectomy | 45.8 |
| CPT-4 procedure codes | |
| Colectomy, partial; with anastomosis | 44140 |
| Colectomy, partial; with skin level cecostomy or colostomy | 44141 |
| Colectomy, partial; with end colostomy and closure of distal segment (Hartmann type procedure) | 44143 |
| Colectomy, partial; with resection, with colostomy or ileostomy and creation of mucofistula | 44144 |
| Colectomy, partial; with coloproctostomy (low pelvic anastomosis) | 44145 |
| Colectomy, partial; with coloproctostomy (low pelvic anastomosis), with colostomy | 44146 |
| Colectomy, partial; abdominal and transanal approach | 44147 |
